# The Erasmus Glioma Database (EGD): Structural MRI scans, WHO 2016 subtypes, and segmentations of 774 patients with glioma

**DOI:** 10.1016/j.dib.2021.107191

**Published:** 2021-06-02

**Authors:** Sebastian R. van der Voort, Fatih Incekara, Maarten M.J. Wijnenga, Georgios Kapsas, Renske Gahrmann, Joost W. Schouten, Hendrikus J. Dubbink, Arnaud J.P.E. Vincent, Martin J. van den Bent, Pim J. French, Stefan Klein, Marion Smits

**Affiliations:** a Biomedical Imaging Group Rotterdam, Department of Radiology and Nuclear Medicine, Erasmus MC University Medical Centre Rotterdam, Rotterdam, the Netherlands; b Department of Radiology and Nuclear Medicine, Erasmus MC University Medical Centre Rotterdam, Rotterdam, the Netherlands; c Department of Neurosurgery, Brain Tumor Center, Erasmus MC University Medical Centre Rotterdam, Rotterdam, the Netherlands; d Department of Neurology, Brain Tumor Center, Erasmus MC Cancer Institute, Rotterdam, the Netherlands; e Department of Pathology, Brain Tumor Center, Erasmus MC Cancer Institute, Rotterdam, the Netherlands

**Keywords:** Magnetic resonance imaging, Glioma, Radiomics, Segmentation, Genetics, WHO 2016, Brain

## Abstract

The Erasmus Glioma Database (EGD) contains structural magnetic resonance imaging (MRI) scans, genetic and histological features (specifying the WHO 2016 subtype), and whole tumor segmentations of patients with glioma. Pre-operative MRI data of 774 patients with glioma (281 female, 492 male, 1 unknown, age range 19–86 years) treated at the Erasmus MC between 2008 and 2018 is available. For all patients a pre-contrast T1-weighted, post-contrast T1-weighted, T2-weighted, and T2-weighted FLAIR scan are available, made on a variety of scanners from four different vendors. All scans are registered to a common atlas and defaced. Genetic and histological data consists of the IDH mutation status (available for 467 patients), 1p/19q co-deletion status (available for 259 patients), and grade (available for 716 patients). The full WHO 2016 subtype is available for 415 patients. Manual segmentations are available for 374 patients and automatically generated segmentations are available for 400 patients. The dataset can be used to relate the visual appearance of the tumor on the scan with the genetic and histological features, and to develop automatic segmentation methods.

## Specifications Table

**Table:** 

Subject	Medical Imaging, Clinical Genetics
Specific subject area	Structural MRI scans, WHO 2016 subtypes, tumor segmentations of patients with glioma
Type of data	MRI data (NIfTI files):
	Pre-contrast T1-weighted
	Post-contrast T1-weighted
	T2-weighted
	T2-weighted FLAIR
	Genetic and histological data (Excel files):
	IDH mutation status
	1p/19q co-deletion status
	Grade
	Tumor segmentations (NIfTI files)
How data were acquired	MRI Scans were acquired on a variety of scanners and field strengths from four different vendors.
	Genetic and histological data were obtained by analysis of tumor tissue obtained from biopsy or resection.
	Whole tumor segmentations were manually annotated by one of four different observers or automatically generated using a convolutional neural network [1].
Data format	Raw
Parameters for data collection	MRI images were acquired using a range of different settings.
Description of data collection	Patients with glioma treated at the Erasmus MC between 2008 and 2018 were retrospectively included. Pre-operative imaging was acquired according to routine clinical protocols. IDH mutation status, 1p/19q co-deletion status, and grade were determined either as part of the treatment process or for research purposes.
Data source location	Erasmus MC (University Medical Center Rotterdam)
	Rotterdam
	The Netherlands
Data accessibility	Repository name: Health-RI XNAT
	Data identification number: EGD
	Direct URL to data: https://xnat.bmia.nl/data/archive/projects/egd
	The data usage agreement is available as a supplementary file.
	The data downloader is available at https://doi.org/10.5281/zenodo.4761088.

## Value of the Data

• This dataset provides imaging data, genetic and histological data, and outlined tumors from a large number of patients with glioma. Currently, limited data is available that provides all this information for a single patient cohort. Data has been collected from routine clinical care, thus representing the real-life variability of the data. This real-life, heterogenous nature of the data, in combination with its size, makes the dataset a valuable resource.
• This dataset will be beneficial for researchers working on the analysis of glioma based on MRI scans.
• This data can be used to validate or develop radiomics methods and automated segmentation methods. For example, the data can be used as a large, heterogenous independent test set, or to increase the size and heterogeneity of train sets for developing new methods.

## 1 Data Description

The Erasmus Glioma Database (EGD) contains 774 patients with glioma and provides three different sources of data:

1. Structural MRI scans
2. Genetic and histological labels specifying the WHO 2016 subtype
3. Tumor segmentations

Data is available on an XNAT server which allows access to the data through an API [2]. We have also created a Docker image that can be used to download the data locally according to the data structure described in this paper: https://doi.org/10.5281/zenodo.4761088.

### 1.1 Structural MRI scans

For all patients four types of structural MRI scans are available: pre-contrast T1-weighted, post-contrast T1-weighted, T2-weighted, and T2-weighted FLAIR. These scans are provided as NIfTI files named “T1.nii.gz”, “T1GD.nii.gz”, “T2.nii.gz” and “FLAIR.nii.gz”, with one folder per subject containing all four scans. Scans have been converted from DICOM to NIfTI using dcm2niix version v1.0.20190410 [3], and have been registered to the MNI atlas using Elastix version 5.0.0 [4], [5]. An overview of the MRI vendor (DICOM tag (0008, 0070)), scanner model (DICOM tag (0008,1090)), and field strength (DICOM tag (0018,0087)) per patient is provided in the accompanying excel sheet “Scan_characteristics.xlsx”.

Clinical data is provided in the Excel sheet “Clinical_data.xlsx”, which includes the patient age in years at the time of the scan and the patient sex. When the patient age or sex is unknown this is indicated by the label −1.

### 1.2 Genetic and histological labels

**Fig. 1:**
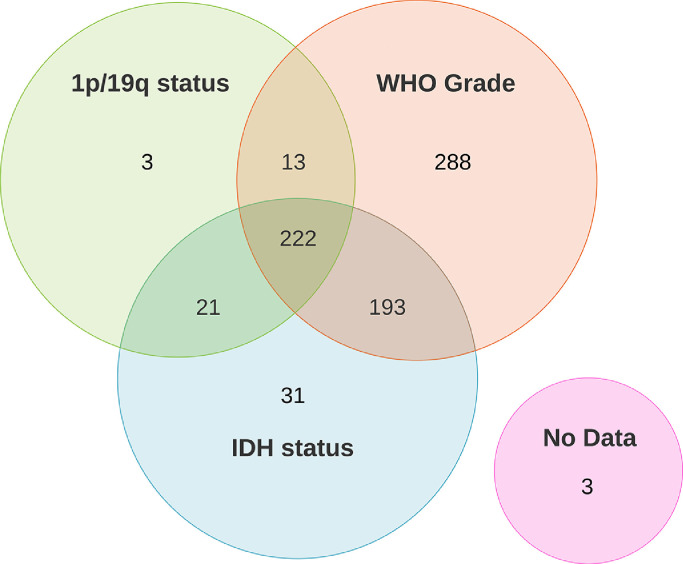
Overview of number of patients with available genetic and histological data and the overlap between different groups. The WHO 2016 subtype is available for the 222 patients for which the IDH mutation status, 1p/19q co-deletion status, and tumor grade is known and for the 193 patients for which the IDH mutation status and tumor grade is known.

Genetic labels consist of the IDH mutation status and the 1p/19q co-deletion status; histological labels consist of the tumor grade. These genetic and histological labels allow for the specification of the WHO 2016 subtype [6]. IDH mutation status is available for 467 patients, 1p/19q co-deletion data is available for 259 patients, and tumor grade is available for 716 patients; a detailed overview is given in Fig. 1. For 222 patients the IDH mutation status, 1p/19q co-deletion status, and tumor grade are all known. For 193 patients the IDH mutation status and grade are known where the tumor was either IDH wildtype or was a grade IV tumor, obviating the need for the 1p/19q co-deletion status to determine the WHO 2016 subtype. Thus, the full WHO 2016 subtype is available for 415 patients.

The genetic and histological labels for each patient are available as an Excel sheet: “Genetic_and_Histological_labels.xlsx”. For the IDH mutation status and the 1p/19q co-deletion status patients have either label 1 (when the tumor was IDH mutated or 1p/19q co-deleted, respectively) or label 0 (when the tumor was IDH wildtype or 1p/19q intact, respectively). Grade is indicated with label 2, 3, or 4 for WHO grade II, III, or IV, respectively. For all cases missing data is indicated by the label −1.

### 1.3 Tumor segmentations

For 374 patients a manually annotated whole tumor segmentation and for 400 patients an automatic whole tumor segmentation is available as “MASK.nii.gz” in the patient subfolder, alongside the four scans. A label of 1 indicates tumor, and a label of 0 indicates background. The manual tumor segmentations were made by one of four different observers based on either the T2-weighted scan or the T2-weighted FLAIR scan. The automatic tumor segmentations were made based on the pre- and post-contrast T1-weighted scan, the T2-weighted scan, and the T2-weighted FLAIR scan using a convolutional neural network (CNN) [1]. This CNN was trained using the manually segmented scans (in addition to other manually segmented scans not included in this data release), therefore, the CNN was not used to create automatic segmentations for the manually segmented scans. The Excel sheet “Segmentation_source.xlsx” provides an overview of the observer of the segmentation, indicated as OBS1, OBS2, OBS3, or OBS4 for the four manual observers or as AUTO if the segmentation was made by the CNN, and the scan that was used as the basis of the segmentation for the manually annotated scans.

The data that is available in the Excel sheets (the scanner data, clinical data, genetic and histological labels, and information about the segmentation) is also available in each patient folder as “metadata.json” to allow for easier automatic processing.

## 2 Experimental Design, Materials and Methods

Data was retrospectively collected for patients with diffuse glioma who were treated at the Erasmus MC, The Netherlands, between 2008 and 2018. Patients were included if they were at least 18 years old and if pre-operative pre-contrast T1-weighted, post-contrast T1-weighted, T2-weighted and T2-weighted FLAIR were available.

### 2.1 Imaging

Scans were retrospectively collected from the imaging that was performed as part of the routine clinical care for each patient. Scans were acquired on scanners from four vendors: Siemens (347 patients), Philips (254 patients), GE (172 patients), and Toshiba (1 patient), using a field strength of 3T (83 patients), 1.5T (571 patients), 1T (110 patients), or 0.5T (6 patients). For 4 patients the field strength was not known.

All scans were registered to the MNI152 atlas, version ICBM 2009a nonlinear, which has a voxel size of 1×1×1 mm${}^{3}$ and a size of 197×233×189 voxels [7], [8]. The scans were affinely registered using Elastix version 5.0.0 [4], [5]. The pre- and post-contrast T1-weighted scans were registered to the T1-weighted atlas; the T2-weighted and T2-weighted FLAIR scans were registered to the T2-weighted atlas. When a manual segmentation was available for a patient, the registration parameters that resulted from registering the scan used during the segmentation were used to transform the segmentation to the atlas. Registration parameters are available at the Elastix Model Zoo under ID Par0064 (https://elastix.lumc.nl/modelzoo/par0064/).

After image registration, the scans were then defaced using a mask that was created based on the MNI atlas; this mask is available as “Deface_mask.nii.gz”. All voxels outside this mask were set to 0, voxels within the mask kept their original value. The defacing mask included as much of the skull as possible, while ensuring the privacy of the patient by removing the characteristic facial features. For future processing of the dataset a brain mask that can be used for skull stripping is also included, available as “Brain_mask.nii.gz”. This brain mask was made using HD-BET [9].

### 2.2 Genetics

Genetic and histological features were determined from tumor tissue that was obtained through biopsy or resection as part of the routine clinical care. Formalin-fixed-paraffin-embedded (FFPE) tissue sections were macro-dissected, selected for areas with highest tumor content as marked by the neuro-pathologist. DNA was isolated from these sections as described by Wijnenga et al. [10]. A dedicated sequencing panel was then used for the molecular analysis to screen for IDH1 or IDH2 mutations and 1p/19q co-deletion [11].

### 2.3 Segmentation

Manual segmentations were made using SimpleITK v3.6.0 [12] or BrainLab (BrainLab, Feldkirchen, Germany, version 2.1.0.15), with which the whole tumor was segmented based on the hyperintensities on either the T2-weighted FLAIR or the T2-weighted scan. Manual segmentations were made based on the scans before registration to the atlas.

Automatic segmentations were based on the registered scans, and were generated using a CNN; for more details see Van der Voort et al. [1].

## Ethics Statement

The study was performed in accordance with the 1964 Helsinki Declaration and its later amendments or comparable ethical standards.

## CRediT Author Statement

**Sebastian R van der Voor:** Conceptualization, Software, Investigation, Data Curation, Writing - Original Draft, Writing - Review & Editing, Visualization; **Fatih Incekara:** Conceptualization, Investigation, Resources, Data Curation, Writing - Review & Editing; **Maarten MJ Wijnenga:** Investigation, Resources, Data Curation, Writing - Review & Editing; **Georgios Kapsas:** Investigation, Resources, Data Curation, Writing - Review & Editing; **Renske Gahrmann:** Investigation, Resources, Data Curation, Writing - Review & Editing; **Joost W Schouten:** Resources, Data Curation, Writing - Review & Editing; **Hendrikus J Dubbink:** Resources, Data Curation, Writing - Review & Editing; **Arnaud JPE Vincent** Resources, Data Curation, Writing - Review & Editing; **Martin J van den Bent:** Resources, Data Curation, Writing - Review & Editing, Supervision; **Pim J French:** Investigation, Resources, Data Curation, Writing - Original Draft, Writing - Review & Editing; **Stefan Klein:** Conceptualization, Writing - Original Draft, Writing - Review & Editing, Supervision, Funding acquisition; **Marion Smits:** Resources, Data Curation, Conceptualization, Writing - Original Draft, Writing - Review & Editing, Supervision, Funding acquisition.

## Declaration of Competing Interest

Hendrikus Dubbink has the following interest that are not related to the current work: grants, personal fees and non-financial support from AstraZeneca, personal fees from AbbVie, personal fees from Janssen, personal fees from Pfizer, personal fees from PGDx, personal fees from MSD, personal fees from Lilly.

Marion Smits received honoraria for independent trial review from Parexel Ltd (paid to institution, no direct relation with the presented work) and speaker fees from GE Healthcare (paid to institution, no direct relation to the presented work).

## Appendix A Supplementary materials

Supplementary Data S1 Supplementary Raw Research Data. This is open data under the CC BY license http://creativecommons.org/licenses/by/4.0/ Supplementary Data S1
